# Malaria in Solid Organ Transplant Recipients: Transmission, Diagnosis, Geographic Patterns, and the Role of Cyclosporin

**DOI:** 10.3390/pathogens15080829

**Published:** 2026-08-06

**Authors:** Wanesa Wilczyńska, Małgorzata Marchelek-Myśliwiec, Krzysztof Korzeniewski, Michał Sławiński, Danuta Kosik-Bogacka

**Affiliations:** 1 Department of Epidemiology and Tropical Medicine, Military Institute of Medicine–National Research Institute, 04-141 Warsaw, Poland; wwilczynska@wim.mil.pl (W.W.); kkorzeniewski@wim.mil.pl (K.K.); 2 Clinic of Nephrology, Transplantology and Internal Medicine, Pomeranian Medical University in Szczecin, Powstańców Wielkopolskich 72, 70-111 Szczecin, Poland; malgorzata.marchelek.mysliwiec@pum.edu.pl; 3 Department of Laboratory Diagnostics, University Clinical Hospital No. 2, Pomeranian Medical University in Szczecin, 72 Powstańców Wlkp. Al., 70-111 Szczecin, Poland; m.slawinski@usk2.szczecin.pl; 4 Department of Biology, Parasitology and Pharmaceutical Botany, Pomeranian Medical University in Szczecin, Powstańców Wielkopolskich 72, 70-111 Szczecin, Poland

**Keywords:** malaria, *Plasmodium* spp., solid organ transplantation, immunosuppression, donor-derived infection

## Abstract

Malaria is one of the most severe parasitic diseases worldwide and presents unique challenges in solid organ transplantation (SOT). Donor-derived malaria, although rare, can cause severe morbidity and mortality due to diagnostic delays and the altered immunity of transplant recipients. This manuscript synthesizes available evidence on transmission routes, diagnostic timing, clinical manifestations, geographic variability, and cyclosporin’s mechanistic antimalarial properties, and integrates recommendations from major transplant associations. The findings highlight that global travel and migration have increased donor-derived malaria risk, Polymerase Chain Reaction (PCR) is the most sensitive screening tool, and cyclosporin shows potent *in vitro* antiplasmodial activity without documented clinical protection. International guidelines consistently emphasize epidemiologic donor screening, risk-based laboratory testing, post-transplant surveillance, and pre-travel prophylaxis.

## 1. Introduction

Malaria remains one of the most devastating parasitic diseases globally, posing a continuous threat to public health in endemic regions and emerging as a distinct complication in modern transplant medicine. Despite significant global efforts toward eradication, the disease remains endemic in 80 countries. According to recent data from the World Health Organization (WHO), approximately 282 million cases of malaria and 610,000 deaths were recorded worldwide in 2024, with the vast majority concentrated in Sub-Saharan Africa. The etiological agents of human malaria include five *Plasmodium* species: *Plasmodium falciparum*, *P. vivax*, *P. malariae*, *P. ovale*, and *P. knowlesi*. In regions with high transmission dynamics, mixed infections are frequently observed; co-infections of *P. falciparum* with *P. malariae* or *P. ovale* predominate in Africa, whereas mixed infections involving *P. falciparum*, *P. vivax*, and *P. knowlesi* are commonly documented in Southeast Asia [1]. In non-endemic countries, malaria is traditionally classified as an imported disease associated with travel or migration. However, the landscape of solid organ transplantation (SOT) has been profoundly altered by globalization, increasing international travel, and migration patterns, particularly involving individuals visiting friends and relatives (VFR) [2,3,4]. These shifting demographics have inadvertently introduced latent or asymptomatic *Plasmodium* spp. carriers into the organ donor pools of non-endemic nations. Consequently, donor-derived malaria infection (DDI) is increasingly recognized as a rare but potentially fatal complication across kidney, liver, heart, and lung transplantation programs [2,5]. While natural transmission occurs via the bite of an infected female *Anopheles* mosquito, transplant recipients can acquire the parasite directly through the allograft tissue or via contaminated blood product transfusions. Managing malaria in the SOT population presents exceptional clinical challenges. The necessity of chronic maintenance immunosuppressive therapy profoundly alters the host immune response, leading to atypical clinical presentations, attenuation of classic inflammatory symptoms, and delayed recognition of infection. These factors contribute to profound diagnostic delays, which significantly increase the risk of severe hemolytic anemia, thrombocytopenia, multi-organ failure, and graft dysfunction [2,5,6]. Therefore, this review aims to synthesize current knowledge regarding the epidemiology, transmission routes, complex diagnostic challenges, and international screening guidelines for malaria in SOT recipients. Furthermore, we investigate the dual nature of cyclosporin A (CsA) —analyzing its well-documented preclinical antiplasmodial properties against its lack of verified clinical protection in real-world transplant settings.

## 2. Literature Search

### 2.1. Search Strategy and Information Sources

To ensure a comprehensive and transparent synthesis of available evidence, a structured literature review was conducted. Comprehensive electronic searches were systematically performed across major peer-reviewed biomedical databases, including PubMed, the National Center for Biotechnology Information (NCBI) repository, ScienceDirect, and Google Scholar. The search architecture was designed to capture all relevant publications bridging parasitology, tropical medicine, pharmacology, and SOT.

The search string was constructed using medical subject headings (MeSH) and specific free-text keywords combined with Boolean operators (AND, OR). The primary search terms included: “malaria”, “*Plasmodium* spp.”, “solid organ transplantation”, “SOT”, “donor-derived infection”, “DDI”, “immunosuppression”, “cyclosporine A”, “tacrolimus”, “kidney transplantation”, “liver transplantation”, “heart transplantation”, “lung transplantation”, “chemoprophylaxis”, and “transplant tourism”.

### 2.2. Inclusion Criteria and Study Selection

Publications were evaluated and selected based on their methodological rigor and relevance to the scope of this review. The inclusion criteria comprised: i/ peer-reviewed original research articles, prospective and retrospective clinical studies, and multi-center cohort analyses; ii/ detailed clinical case reports and literature reviews documenting laboratory-confirmed post-transplant malaria or donor-derived transmission clusters; iii/ official guidelines, consensus statements, and policy recommendations published by recognized international transplantation and infectious disease societies (e.g., the American Society of Transplantation—AST, and the World Health Organization—WHO) and iv/ preclinical *in vitro* and *in vivo* studies investigating the molecular, biochemical, and pharmacological antiplasmodial mechanisms of CsA and its chemical analogs.

The initial search scope targets literature spanning from 1978 to 2026, allowing for both a historical evaluation of early documented transmission cases and the integration of contemporary molecular data and emerging epidemiological registries up to the current year of 2026. No geographic restrictions were applied. Non-English and non-Polish publications were excluded unless official peer-reviewed translations were available.

### 2.3. Data Extraction and Synthesis

Data from the selected literature were systematically extracted and categorized into four primary thematic streams: (1) epidemiological demographics and organ-specific transmission vectors; (2) clinical and laboratory presentations with special emphasis on the confounding effects of immunosuppression; (3) the comparative diagnostic performance of microscopy, antigen-detecting rapid tests, and PCR assays; and (4) the pharmacological safety profiles and critical drug–drug interactions of conventional antimalarials. The extracted case registries were consolidated into a comprehensive reference database (presented in Appendix A Table A1) to evaluate recipient clinical outcomes and formulate standardized recommendations.

## 3. Donor-Derived Malaria in SOT

### 3.1. Mechanisms and Routes of Transmission

Donor-derived malaria occurs when latent, submicroscopic, or asymptomatic parasitemia present in an organ donor is inadvertently transmitted to the recipient through the transplanted organ or residual donor blood [5,7]. This represents an important biovigilance challenge because experimental studies have demonstrated that intraerythrocytic *P. falciparum* parasites remain viable during refrigerated storage, indicating that low-temperature preservation alone is insufficient to eliminate the risk of transmission [8,9]. Clinical reports of donor-derived malaria further confirm that standard organ preservation procedures do not reliably prevent parasite transmission [2,5,10]. Concurrently, the risk of reactivation becomes particularly relevant when a recipient harboring a dormant malaria parasite receives immunosuppressant drugs, as immunosuppressive therapy may activate latent infection and lead to clinical malaria [5,10].

Globalization, migration, and international travel significantly expand the number of donors with malaria exposure. Immigrants and travelers (VFR) contribute disproportionately to imported malaria cases. These exposures often go unrecognized during donor interviews, resulting in inadequate screening [3,4].

**Figure 1:**
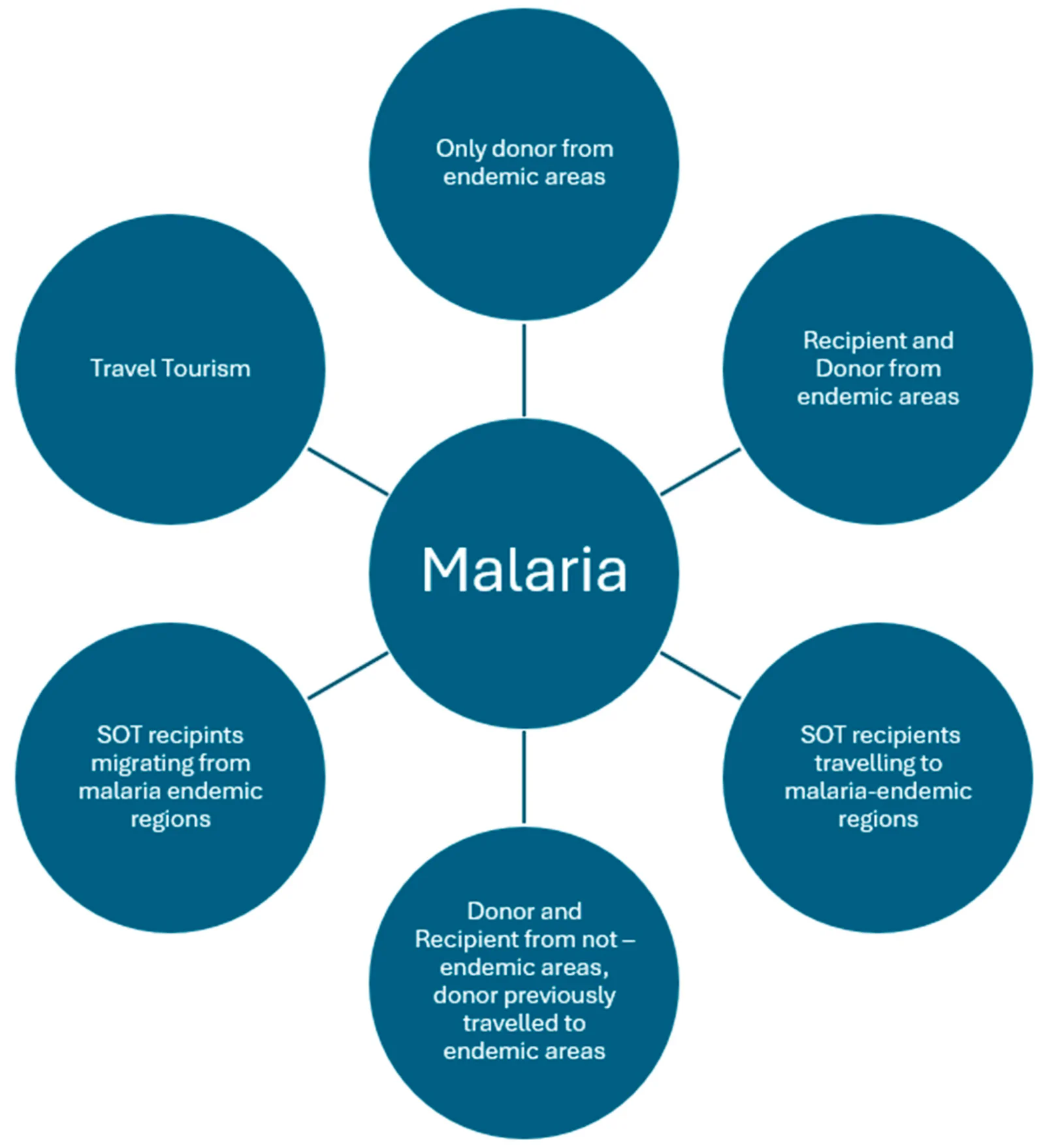
Epidemiological scenarios of malaria acquisition in SOT recipients.

Based on the published literature on donor-derived, imported, and post-transplant malaria, six epidemiological scenarios of malaria acquisition in SOT can be distinguished (Figure 1). The proposed classification was developed by the authors based on synthesis of published case reports and review articles summarized in Appendix A.

1. Endemic Co-localization: Situations where both the donor and the recipient permanently reside in a malaria-endemic geographic region and the transplantation is performed locally.

2. Endemic Donor Migration: Cases where the recipient resides in a non-endemic country, but the organ originates from a donor who migrated from a highly endemic region.

3. Recipient Travel/Tourism: SOT recipients from non-endemic areas who acquire de novo infection while traveling to malaria-endemic regions for leisure or tourism.

4. Donor Travel History: Scenarios where both the donor and recipient live in a non-endemic region, but the donor had a historical travel exposure to an endemic area, harboring asymptomatic parasitemia.

5. Recipient Migration: SOT recipients who originally migrated from endemic areas to non-endemic countries and experience post-transplant reactivation or relapse due to therapeutic immunosuppression. Immunosuppressive therapy may activate latent parasites harbored by the patient, transforming asymptomatic or dormant parasitemia into clinical malaria.

6. Transplant Tourism: Individuals from non-endemic regions who deliberately travel to foreign endemic areas to undergo commercial living-donor organ transplantation—most frequently traveling to India for liver fragments or Pakistan and China for kidney transplants.

**Figure 2:**
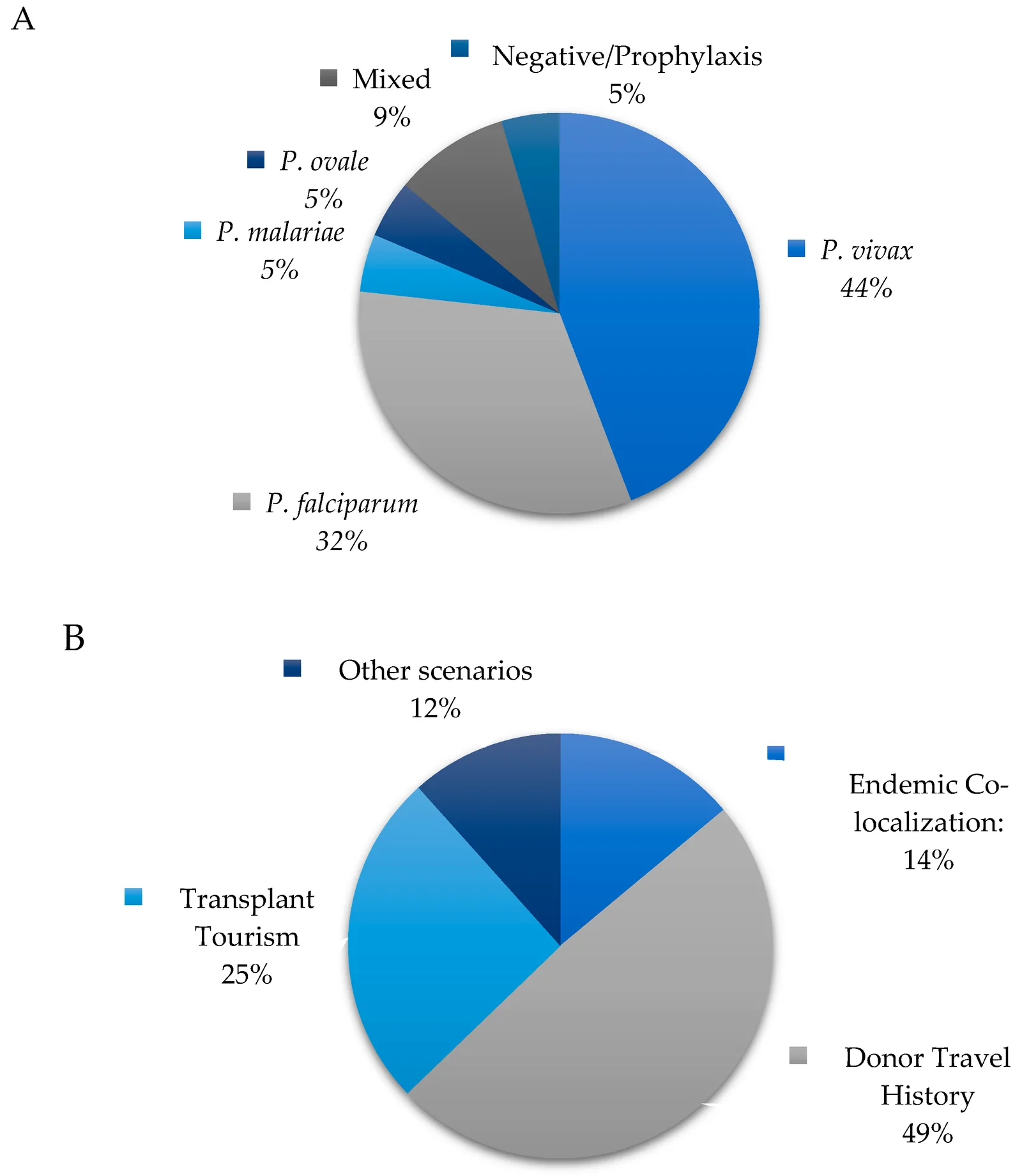
Quantitative analysis of SOT-associated malaria cases from the pooled cohort. (**A**) Distribution of cases by *Plasmodium* species, highlighting the predominance of *P. vivax* and *P. falciparum*. (**B**) Breakdown of documented cases according to the 6 predefined epidemiological scenarios, showcasing the critical impact of asymptomatic donor travel history and transplant tourism.

A detailed metrics analysis of the pooled cohort ((*n* = 43) recipients) revealed distinct species-specific and epidemiological patterns. Regarding species distribution (Figure 2A), *P. vivax* was the most prevalent pathogen, accounting for 44.2% (19/43) of cases, closely followed by *P. falciparum* at 32.6% (14/43). Mixed infections (primarily *P. falciparum* and *P. vivax*) constituted 9.3% (4/43), while *P. malariae* and *P. ovale* remained rare, each representing 4.7% (2/43) of the cohort.

When mapped against the proposed epidemiological frameworks (Figure 2B), Scenario 4 (Donor Travel History/Asymptomatic Carrier in non-endemic countries) emerged as the leading driver of transmission, encompassing 48.8% (21/43) of cases. This was followed by Scenario 6 (Transplant Tourism) at 25.6% (11/43), and Scenario 1 (Endemic Co-localization) at 14.0% (6/43). Notably, the cohort analysis underscored the phenomenon of ‘transmission clusters’, where a single asymptomatic multi-organ donor unknowingly transmitted the parasite to up to four different recipients, demonstrating the profound systemic risk of single-donor screening omissions.

### 3.2. Organ-Specific Distribution and Clinical Outcomes

Analysis of the published cases summarized in Appendix A Table A1 demonstrates that the clinical presentation and outcomes of donor-derived malaria vary according to the transplanted organ. Although the overall number of reported cases remains limited, several consistent patterns emerge regarding disease presentation, diagnostic challenges, and clinical outcomes (Table 1).

The observations presented below are based on the analysis of all published cases identified in the literature and summarized in Appendix A, supplemented by recent review articles.

Kidney transplantation accounts for the largest proportion of published donor-derived malaria cases, likely reflecting the high global volume of renal transplantation and the greater use of living donors. Most reported recipients developed fever accompanied by acute kidney injury, thrombocytopenia, anemia, and varying degrees of graft dysfunction. Importantly, the majority of patients recovered following timely diagnosis and initiation of species-appropriate antimalarial therapy [2,5,11,12].

Liver transplant recipients represent the second largest group of reported cases. A critical, yet frequently overlooked biological hazard in liver transplantation is the management of hypnozoites—the latent liver-stage forms characteristic of *P. vivax* and *P. ovale*. Hypnozoites can remain dormant within host hepatocytes for months or even years, successfully evading standard blood-screening modalities like peripheral blood microscopy or rapid diagnostic tests (RDTs). In the context of liver transplantation, an asymptomatic donor carrying dormant hypnozoites will directly transfer the intrahepatic parasite reservoir within the allograft tissue to the recipient. Following transplantation, the mandatory initiation of potent immunosuppressive regimens serves as a major trigger for the reactivation of these dormant forms. This leads to de novo erythrocytic cycles and acute post-transplant malaria [5,6,13,14].

Heart transplantation has been associated with only a limited number of published cases; however, several reports describe rapidly progressive disease characterized by circulatory collapse, severe metabolic disturbances, and neurological complications. Although firm conclusions cannot be drawn because of the small number of observations, the available literature suggests that donor-derived malaria following heart transplantation may present with a particularly severe clinical course [2,14,15].

Lung transplantation remains the least frequently reported setting for donor-derived malaria. Published cases demonstrate that the initial presentation is often nonspecific, emphasizing the importance of epidemiological risk assessment and early laboratory investigation. Despite occasional diagnostic difficulties, including false-negative donor screening results, favorable outcomes have generally been achieved following prompt diagnosis and treatment [16,17].

## 4. Clinical Manifestations and the Immunological Impact of Immunosuppression

### 4.1. Pathophysiological Alterations and Cytokine Suppression

In immunocompetent individuals, the erythrocytic stage of *Plasmodium* spp. infection triggers a robust innate immune response characterized by the synchronized release of pyrogenic pro-inflammatory cytokines, which clinically manifests as paroxysmal fever, chills, and rigors. However, the mandatory maintenance immunosuppressive regimens administered to SOT recipients fundamentally alter this classical pathophysiological cascade [7,18,19] (Pierrotti et al., 2018; Schwartz, 2013; Wołyniec et al., 2018).

Therapeutic agents frequently utilized in SOT induction and maintenance—including anti-thymocyte globulin (ATG), calcineurin inhibitors (tacrolimus, cyclosporine), and antimetabolites (mycophenolate mofetil, azathioprine)—profoundly suppress T-lymphocyte function and disrupt downstream immune signaling pathways essential for effective parasite clearance [7,18]. Calcineurin inhibitors directly interfere with T-cell activation and cytokine transcription, while antiproliferative agents reduce clonal expansion of effector lymphocytes, collectively leading to impaired coordination of the host immune response.

At the molecular level, these immunosuppressive regimens reduce the production of key pro-inflammatory cytokines, including Interleukin-1 (IL-1), Interleukin-6 (IL-6), Interferon-gamma (IFN-γ), and Tumor Necrosis Factor-alpha (TNF-α), thereby attenuating systemic inflammatory signaling [18,19]. Because TNF-α and IL-1 serve as primary endogenous pyrogens acting on the hypothalamic thermoregulatory center, their suppression contributes to an attenuated or atypical febrile response.

Consequently, SOT recipients may present with delayed diagnosis or atypical, often afebrile clinical courses of malaria despite significant parasitemia, as the classical fever-paroxysm pattern is blunted or absent under immunosuppressive therapy [5,7,10,19]. This diagnostic masking effect has been repeatedly described in clinical case series and reviews of post-transplant malaria.

### 4.2. Clinical Consequences and Graft End-Organ Damage

The blunted immunological response in SOT recipients facilitates uncontrolled intracellular replication of *Plasmodium* spp. within erythrocytes, resulting in increased parasite burden. This accelerated replication cycle contributes to enhanced intravascular hemolysis, leading to acute hemolytic anemia and thrombocytopenia, which are well-documented complications in transplant-associated malaria [5,18,19].

Beyond systemic hematological involvement, transplant-transmitted malaria may cause direct microvascular and allograft-specific injury. In *P. falciparum* infection, parasite-induced surface adhesins expressed on infected erythrocytes mediate binding to endothelial receptors, resulting in cytoadherence and sequestration within the microcirculation [5,18]. This phenomenon leads to obstruction of capillary blood flow and impaired tissue perfusion, which is particularly relevant in highly vascularized transplanted organs.

Microvascular sequestration results in localized ischemia and tissue hypoxia, contributing to organ dysfunction. In renal allografts, malaria-associated microcirculatory impairment may present with acute kidney injury, including acute tubular injury and features overlapping with thrombotic microangiopathy [18,19,20]. In hepatic grafts, parasite-induced hemolysis and intrahepatic microvascular dysfunction can contribute to hyperbilirubinemia and elevated liver enzymes, reflecting combined hemolytic and hepatocellular injury [2].

Importantly, the clinical and laboratory presentation of graft dysfunction in malaria-infected SOT recipients may closely mimic acute allograft rejection. Overlapping features such as graft tenderness, rising creatinine or liver enzymes, and systemic inflammatory response can lead to diagnostic confusion [18,19]. Misinterpretation of these findings as rejection may result in escalation of immunosuppressive therapy (e.g., high-dose corticosteroids or anti-thymocyte globulin), which further suppresses host immune control and may exacerbate parasitemia, increasing the risk of rapid clinical deterioration and multi-organ failure [2,18,19].

## 5. Diagnostic Challenges and Modalities in the SOT Population

### 5.1. Comparative Performance of Laboratory Techniques

Establishing a timely diagnosis of *Plasmodium* spp. infection in SOT recipients is challenging due to low clinical suspicion in non-endemic settings and overlapping clinical features with other post-transplant complications. Accurate laboratory identification requires sensitive diagnostic tools capable of detecting low-level parasitemia, which may occur in asymptomatic donor-derived infections or early-stage disease [2,5,18].

#### 5.1.1. Blood-Smear Microscopy (Gold Standard)

Light microscopy of thick and thin peripheral blood films remains the reference standard for malaria diagnosis, enabling species identification, quantification of parasitemia, and assessment of treatment response. However, its sensitivity decreases significantly at low parasite densities, typically below 50–200 parasites/µL. In SOT recipients, where clinical presentation may be atypical or attenuated, reliance on microscopy alone may contribute to delayed diagnosis. In addition, microscopy is highly operator-dependent and requires experienced personnel, which may not be available in non-endemic transplant centers [18,19].

#### 5.1.2. Rapid Diagnostic Tests

Antigen-based rapid diagnostic tests provide a rapid and accessible screening tool for malaria detection. Most commercially available assays target histidine-rich protein 2 (HRP2), which is highly specific for *P. falciparum*. Reported sensitivity for *P. falciparum* approaches approximately 90%, while performance is lower for non-falciparum species such as *P. vivax* [5]. However, the emergence of *P. falciparum* strains with pfhrp2/pfhrp3 gene deletions has reduced test reliability and may lead to false-negative results. Conversely, persistent antigenemia following parasite clearance may result in false-positive results, limiting their utility for treatment monitoring. Tests targeting parasite lactate dehydrogenase (pLDH) partially overcome HRP2-related limitations but remain less sensitive in low-parasitemia infections [2,5].

In malaria-endemic regions where solid organ transplantation is performed, selecting the appropriate RDT design is vital. While HRP2 is a highly sensitive biomarker, its utility is strictly limited to detecting *P. falciparum*. To effectively diagnose non-falciparum species—such as *P. vivax* in South America and Asia, or *P. ovale* and *P. malariae* in Africa—RDTs must incorporate alternative biomarkers, specifically pLDH and aldolase. Aldolase acts as a pan-malarial marker present in all five human-infecting species. Similarly, species-specific pLDH assays allow differentiation between *P. falciparum* and non-falciparum infections. Utilizing multi-antigen RDTs (e.g., HRP2/pLDH combos) is imperative in SOT surveillance to prevent false negatives in patients infected with non-falciparum strains, which could otherwise lead to delayed treatment and subsequent graft loss [2,5].

#### 5.1.3. Molecular Diagnostics (PCR/NAAT)

PCR and other nucleic acid amplification techniques provide the highest sensitivity and specificity for malaria detection, including submicroscopic infections with parasite densities below 1 parasite/µL. These methods are particularly valuable for screening of donors and high-risk transplant candidates from endemic regions [2,21]. Automated hematology analyzers, such as Sysmex XN-series platforms, may incidentally detect malaria-associated abnormalities in leukocyte scattergrams, prompting confirmatory molecular testing [13]. Despite their superior diagnostic performance, molecular methods are limited by cost, infrastructure requirements, and longer turnaround times. Additionally, in transplant recipients, interpretation may be complicated by persistence of circulating parasite nucleic acids after clinical resolution of infection [2,18].

### 5.2. Differential Diagnosis and Clinical Mimicry

Due to the non-specific nature of hematological and biochemical abnormalities in post-transplant malaria, the infection frequently mimics alternative, more common post-operative complications [2,18,19]. To prevent fatal clinical mismanagement, clinicians must maintain a high index of suspicion and recognize the overlapping phenotypic features summarized in Table 2.

## 6. Cyclosporin A: Molecular Mechanisms and the Clinical Paradox

Cyclosporin A, although primarily used as a cornerstone immunosuppressive agent in SOT, has demonstrated intrinsic antiplasmodial activity in experimental studies. Preclinical evidence indicates that CsA inhibits *Plasmodium* growth through multiple mechanisms, including inhibition of parasite cyclophilins and peptidyl-prolyl cis-trans isomerase activity, interference with calcineurin-dependent signaling, disruption of parasite membrane integrity, and modulation of multidrug resistance-associated transporters, including PfMDR1 [22]. Despite these promising *in vitro* and experimental findings, no clinically significant protective effect against malaria has been demonstrated in transplant recipients [18].

The discrepancy between experimental and clinical observations is likely explained by several factors, including the stage-specific activity of CsA against erythrocytic parasites, the inability to achieve antimalarial drug concentrations without unacceptable toxicity, and the dominant immunosuppressive effects of CsA, which impair host immune responses required for parasite control [18,22]. Consequently, current evidence does not support reliance on CsA as a clinically effective antimalarial agent, and standard malaria chemoprophylaxis remains essential for transplant recipients regardless of the immunosuppressive regimen used [18,23].

## 7. International Guidelines, Chemoprophylaxis, and Drug–Drug Interactions

Therapeutic strategies for SOT-associated malaria must be strictly species-specific, accounting for drug resistance patterns and life-cycle biology. For uncomplicated non-falciparum malaria (*P. vivax*, *P. ovale*, *P. malariae*, *P. knowlesi*), artemisinin-based combination therapies (ACTs) or chloroquine (in chloroquine-sensitive regions) remain the standard first-line choice. However, for *P. vivax* and *P. ovale*, standard blood-stage treatment must be combined with radical anti-relapse therapy using primaquine or tafenoquine to eradicate dormant hypnozoites, implemented only after ruling out G6PD deficiency. Conversely, uncomplicated *P. falciparum* requires prompt initiation of ACTs (e.g., artemether-lumefantrine) due to widespread chloroquine resistance. For severe malaria caused by any species (predominantly *P. falciparum*), immediate intravenous artesunate is mandatory, followed by a full oral course of an ACT once the patient can tolerate oral medication [7,23,24].

International transplant societies recommend systematic assessment of malaria risk in both donors and recipients. Donor evaluation should include a detailed epidemiological history focusing on residence in, migration from, or travel to malaria-endemic regions. In donors with epidemiological risk factors, laboratory investigations—including microscopy, serology, and nucleic acid amplification tests (NAATs)—may be considered to identify current or previous exposure, although no single diagnostic method completely excludes malaria infection. Recipients of organs from potentially exposed donors should undergo enhanced clinical surveillance, and malaria should be included in the differential diagnosis of unexplained febrile illness after transplantation [2,10,18,23].

SOT recipients travelling to malaria-endemic areas should receive individualized pre-travel counselling and appropriate chemoprophylaxis [3,23]. Atovaquone–proguanil is generally considered the preferred prophylactic agent because of its favorable safety profile and minimal clinically relevant interactions with calcineurin inhibitors and mammalian target of rapamycin (mTOR) inhibitors [18,23]. Doxycycline represents an effective alternative with similarly limited pharmacokinetic interactions, although its propensity to induce photosensitivity may be particularly relevant in transplant recipients, who already have an increased risk of cutaneous malignancies associated with chronic immunosuppression [23]. Mefloquine may be used in selected patients, but its neuropsychiatric adverse effects and potential to prolong the QT interval require careful risk assessment [18,23]. Primaquine and tafenoquine require prior screening for glucose-6-phosphate dehydrogenase (G6PD) deficiency because of the risk of drug-induced hemolysis [7,24].

Management of malaria in transplant recipients is further complicated by clinically relevant drug–drug interactions between antimalarial agents and immunosuppressive therapies. Chloroquine, quinine, and several other antimalarial drugs may alter the pharmacokinetics of calcineurin inhibitors and mTOR inhibitors, increasing the risk of nephrotoxicity, neurotoxicity, and other treatment-related adverse events [18,25]. Accordingly, therapeutic drug monitoring and dose adjustment of immunosuppressive agents are recommended whenever antimalarial therapy is initiated [18,23]. Additional caution is warranted when antifolate antimalarial drugs are co-administered with trimethoprim–sulfamethoxazole prophylaxis because of the increased risk of clinically significant myelosuppression [18].

## 8. Discussion and Current Knowledge Gaps

Although malaria remains an uncommon complication of SOT in non-endemic countries, its clinical significance is substantial because delayed diagnosis may result in rapid disease progression, graft dysfunction, and increased mortality in immunosuppressed recipients [2,5,18]. This review highlights that malaria in transplant recipients represents a distinct clinical entity in which epidemiological exposure, altered immune responses, atypical clinical manifestations, and complex pharmacological interactions collectively influence patient outcomes.

One of the greatest diagnostic challenges is that *Plasmodium* spp. infection may mimic other common post-transplant complications. Acute deterioration of liver or kidney allograft function, thrombocytopenia, anemia, elevated inflammatory markers, or hepatic injury may initially be attributed to acute rejection, opportunistic infections, drug toxicity, or surgical complications rather than parasitic infection [2,18,19]. In particular, malaria-associated liver injury (MALID) may closely resemble acute liver allograft rejection or drug-induced hepatotoxicity, while acute kidney injury may be indistinguishable from calcineurin inhibitor nephrotoxicity or rejection [2,20,26]. Such diagnostic uncertainty may inadvertently lead to escalation of immunosuppressive therapy before malaria is recognized, potentially facilitating parasite replication and worsening clinical outcomes [18]. Consequently, malaria should remain in the differential diagnosis of unexplained graft dysfunction, especially in recipients or donors with relevant epidemiological risk factors [10,18,23].

Another important challenge is the atypical clinical presentation of malaria in immunosuppressed patients. The classical paroxysmal fever pattern may be absent or poorly developed, whereas organ dysfunction, cytopenias, or nonspecific systemic manifestations may predominate [18,19]. Since malaria is encountered infrequently in most transplant centers outside endemic regions, delayed diagnosis often reflects a low index of clinical suspicion rather than limited access to diagnostic methods. Early recognition therefore depends on careful epidemiological assessment combined with prompt laboratory investigation using microscopy, rapid diagnostic tests, and molecular assays where available [2,5,18,21].

Several important knowledge gaps remain. First, no dedicated European guidelines currently address malaria prevention, diagnosis, or management specifically in SOT recipients. Clinical practice therefore relies on recommendations from international expert groups and national tropical medicine guidelines, resulting in variability in donor screening, recipient evaluation, and post-transplant surveillance among transplant centers [18,23]. Second, although molecular methods demonstrate excellent analytical sensitivity, their diagnostic performance and optimal implementation have not been systematically evaluated in transplant recipients, and current recommendations are largely extrapolated from immunocompetent populations [13,21]. Third, despite convincing experimental evidence demonstrating intrinsic antiplasmodial activity of CsA, no clinical studies have demonstrated a protective effect in transplant recipients, emphasizing the disconnect between preclinical observations and real-world clinical practice [18,22]. Finally, evidence from malaria-endemic low- and middle-income countries remains limited, despite the gradual expansion of transplantation programs in these settings, highlighting the need for internationally representative prospective studies [27,28,29].

## 9. Limitation

While this review provides a comprehensive overview of donor-derived malaria in transplant recipients, several methodological and contextual limitations must be acknowledged:

Due to the extreme rarity of donor-derived malaria, the analyzed literature consists primarily of isolated case reports and small case series rather than large-scale cohort studies. This inherently limits the generalizability of the statistical trends and clinical outcomes described. Furthermore, the antimalarial efficacy of cyclosporine has been demonstrated strictly *in vitro*; currently, there is a lack of robust *in vivo* clinical evidence to confirm this protective effect in human transplant patients. Finally, the high heterogeneity in institutional transplantation protocols across different countries complicates the standardization of our conclusions.

Furthermore, the true incidence of donor-derived malaria may be underestimated due to publication bias and underreporting, particularly in cases with mild or self-limiting clinical presentations. Additionally, older case reports included in this review may not reflect modern diagnostic capabilities, potentially failing to account for the high sensitivity of contemporary molecular assays. Even at present, a significant geographic disparity exists regarding diagnostic access; while PCR is recognized as the gold standard for screening, it remains unavailable in many transplant centers worldwide.

This review is constrained by the inconsistency of international clinical guidelines, which often vary significantly depending on local epidemiological settings and regional transplant society policies. Lastly, global migration patterns and travel trends evolve at a pace that vastly outstrips the publication of official medical statistics, meaning some underlying epidemiological data may not fully capture real-time shifts in malaria transmission risks.

## 10. Recommendations

Transplant physicians must remain highly vigilant for malaria in SOT recipients presenting with unexplained febrile illness, especially given the rise in international travel, migration, and organ sharing.

Centers should implement comprehensive donor risk assessments and utilize advanced laboratory investigations, including molecular diagnostics (PCR) whenever available. Management must focus on the prompt initiation of appropriate antimalarial therapy combined with strict therapeutic drug monitoring (TDM) of immunosuppressive agents to prevent graft rejection or toxicity.

International working groups (e.g., ESGMIT or AST-IDCOP) should collaborate to develop dedicated, evidence-based guidelines specifically for malaria in the SOT population.

Prospective clinical trials are needed to systematically evaluate and validate the performance, timing, and cost-effectiveness of molecular methods in immunosuppressed patients.

Future research must focus on multicenter, internationally representative prospective studies, with a specific emphasis on capturing robust data from expanding transplant programs in low- and middle-income malaria-endemic countries.

## 11. Conclusions

Donor-derived and post-transplant malaria remain rare but potentially life-threatening complications of SOT. As international travel, migration, and organ sharing continue to increase, transplant physicians should maintain a high index of suspicion for malaria in recipients presenting with unexplained febrile illness or compatible epidemiological risk factors. Comprehensive donor risk assessment, appropriate laboratory investigation—including molecular diagnostics where available—prompt initiation of antimalarial therapy, and careful therapeutic drug monitoring of immunosuppressive agents remain the cornerstones of successful management.

Future progress will require harmonized international recommendations, prospective multicenter studies evaluating diagnostic strategies and treatment outcomes in transplant recipients, and improved surveillance from malaria-endemic regions. Addressing these evidence gaps will strengthen transplantation safety and support the development of evidence-based approaches for preventing and managing malaria in this uniquely vulnerable patient population.

## Figures and Tables

**Table 1:** Clinical characteristics of donor-derived malaria according to transplanted organ.

Transplanted Organ	Frequency Among Published Cases *	Typical Clinical Manifestations	Major Diagnostic Challenges	Clinical Outcome
Kidney	Most frequently reported	Fever, acute kidney injury, thrombocytopenia, anemia, graft dysfunction	Differentiation from acute rejection, calcineurin inhibitor nephrotoxicity, bacterial sepsis	Generally favorable following early diagnosis and appropriate antimalarial therapy
Liver	Second most frequently reported	Fever, hepatic dysfunction, thrombocytopenia, hyperbilirubinemia	Distinguishing malaria-associated liver injury from acute rejection, biliary complications, or drug-induced hepatotoxicity	Variable; delayed diagnosis may contribute to severe disease
Heart	Rare	Fulminant malaria, hypotension, metabolic acidosis, neurological complications	Rapid clinical deterioration with overlap with septic shock or primary graft dysfunction	Severe disease reported in several cases, including fatalities
Lung	Very rare	Fever, anemia, nonspecific systemic symptoms	Low clinical suspicion and nonspecific presentation	Favorable when recognized early and treated promptly

* Based on analysis of the published cases summarized in Appendix A Table A1.

**Table 2:** Differential diagnostic framework and clinical mimicry of malaria in SOT recipients.

Clinical/Laboratory Presentation in SOT	Traditional Clinical Interpretation	Actual Malaria Pathophysiology	Confirmatory Diagnostic Action	Ref.
Absence of Fever/Mild Hypothermia	Normal post-operative course; absence of systemic infectious processes.	Iatrogenic suppression of pyrogenic cytokines (IL-1, IL-6, TNF-α) masks the hypothalamic response.	Urgent quantitative PCR/NAAT screening for high-risk epidemiological history.	[18,19]
Allograft Dysfunction (Elevated creatinine/aminotransferases/GGT)	Acute cellular rejection; drug-induced nephrotoxicity or hepatotoxicity	Cytoadherence and sequestration of parasitized erythrocytes in graft capillaries, causing tissue hypoxia and ischemia.	Peripheral thick and thin blood films; allograft biopsy (revealing intravascular parasite crowding).	[2,5,18]
Rapid Cytopenia (Severe hemolytic anemia and thrombocytopenia)	Bone marrow suppression from antimetabolites; cytomegalovirus (CMV) or EBV reactivation.	Accelerated intravascular hemolysis driven by merozoite rupture; splenic sequestration and immune-mediated destruction of platelets.	Reticulocyte count, haptoglobin levels, and sequential blood smears; molecular viral PCR panels.	[18,20]
Encephalopathy and Seizures (PRES-compatible MRI findings)	Cyclosporine/Tacrolimus neurotoxicity; central nervous system bacterial sepsis.	Cerebral malaria pathogenesis; microvascular clogging of cerebral capillaries by parasitized red blood cells.	Fundoscopic examination (malarial retinopathy); immediate thin blood film and urgent antimalarial therapy initiation.	[2,18]

## Data Availability

The analyzed data presented in this study are available in Appendix A.

## Abbreviations

The following abbreviations are used in this manuscript:

AKI	Acute Kidney Injury
AL	Artemether-Lumefantrine
ALT	Alanine Aminotransferase
AST	Aspartate Aminotransferase
ATG	Antithymocyte Globulin
CMV	Cytomegalovirus
DDI	Donor-Derived Infection
DNA	Deoxyribonucleic Acid
EBV	Epstein–Barr Virus
G6PD	Glucose-6-Phosphate Dehydrogenase
Hb	Hemoglobin
ICU	Intensive Care Unit
MMF	Mycophenolate Mofetil
MODS	Multiple Organ Dysfunction Syndrome
PCR	Polymerase Chain Reaction
PLT	Platelet Count
RBC	Red Blood Cell
RNA	Ribonucleic Acid
SOT	Solid Organ Transplantation
Tx	Transplantation
WBC	White Blood Cell

## Appendix A

**Table A1:** Transplant-transmitted malaria (ESKD, Nephropathy progressed to end-stage kidney disease, TAC, tacrolimus; PRED, prednisone; MMF, mycophenolate mofetil; bid, twice daily; AS, Artesunate; AL, artemether–lumefantrine).

Transplant Site	Infection Mode	Donor	Recipient	Post-Transplant Treatment	Clinical Manifestation in the Recipient	Diagnosis	Time from Transplant/Time from Admission to Diagnosis	Species (Parasitaemia)	Treatment	Treatment Outcome	Ref.
**Kidney**											
Saudi Arabia	Transplant Tourism	Living unrelated donor (Pakistan)	52-year-old man from Saudi Arabia, hypertension and type 2 diabetes mellitus with retinopathy and ESKD	Three doses of anti-thymocyte globulin (ATG), tacrolimus, prednisone, and mycophenolate mofetil	Thrombocytopenia, gastrointestinal symptoms	Peripheral blood smear	18 days	*P. falciparum* (80,000 parasites/µL)	AS 2.4 mg/kg daily for six days, followed by oral AL; MMF (500 mg bid), TAC remained stable	No data	[11]
	Transplant Tourism	Living unrelated kidney Transplant, no donor information	42-year-old man with type 1 diabetes mellitus, retinopathy, nephropathy, hypertension, end-stage kidney disease, after living-unrelated kidney transplant in China, which failed due to chronic rejection	Mycophenolate mofetil and tacrolimus and was maintained on low-dose corticosteroids alone	Uremic symptoms and severe graft dysfunction (serum creatinine 1007 µmol/L), fever (38 °C) and tachycardia (117 beats/min)	Peripheral blood smear examination	19 days	*P. vivax* (2%)	Intravenous AS (2.4 mg/kg; 192 mg) for 7 days	No data	
Colombia	Donor-derived transmission	22-year-old male patient with a gunshot wound to the brain and brain death from Venezuela	39-year-old male	Cyclosporine, mycophenolate mofetil, and corticosteroids	Diarrhea, high blood pressure, lower limb edema, and dyspnea caused by small efforts, AKI, severe hyperkalemia, and tubular acidosis	Peripheral blood smear, PCR	8 days/30 days	*P. vivax* (128 parasitic forms/µL)	Chloroquine and primaquine for 14 days	Survived	[2]
Colombia	Donor-derived transmission	Young man who suffered a severe penetrating traumatic brain injury caused by a gunshot wound, who was otherwise healthy	39-year-old man	Tacrolimus, mycophenolate mofetil, and prednisone	Fever, chills, and mild thrombocytopenia	Thick blood smear, PCR	14 days/18 days	*P. vivax* (128 parasitic forms/µL)	Chloroquine plus 14 days of primaquine	Survived	[15]
Spain	Infected donor	Deceased 54-year-old female from Equatorial Guinea	Kidney recipient, 57-year-old man from Spain	No data	Fever (38–39 °C), chills, and urinary symptoms	Peripheral blood smear, PCR, rapid malaria test	30 days	*P. malariae*, *P. ovale*	Chloroquine diphosphate (25 mg base/kg in 4 doses over 48 h)	No data	[10]
Spain	Donor-derived infection	No data	Pancreas-kidney recipient	No data	Asymptomatic	Peripheral blood smear	No information	Negative	Prophylactic treatment with atovaquone-proguanil	Survived	[17]
Spain	Donor-derived infection	No data	Kidney recipient	No data	Asymptomatic	Peripheral blood smear	No information	Negative	Prophylactic treatment with atovaquone-proguanil	Survived	
India	de novo, by the mosquito	His father	20-year-old man from India with chronic glomerulonephritis and end-stage renal disease	No data	Fever (40 °C) with chills/rigor, shortness of breath, decreased urine	Peripheral blood smear, rapid diagnostic test	No data	*P. vivax*	Artesunate, doxycycline	No data	[30]
Spain	Donor-derived infection	Born in Bolivia, lived in Columbia	No data	No data	No data	No data	No data	*P. vivax*	No data	Survived	[31]
Italy	Donor-derived infection	30-year-old man from Ghana	No data	No data	No data	No information	No information	*P. falciparum*	Mefloquine	No information	[32]
Netherlands	Donor-derived infection	His brother from Sudan	37-year-old Sudanese man from Netherlands	No data	Acute kidney injury, fever up to 40 °C	Peripheral thick blood smear and quantitative buffy coat (QBC)	11 days/24 days	*P. falciparum*	Atorvaquone-proquanil combination (Malarone ^TM^)	Survived	[33]
France	Donor-derived infection	Man from Togo, often traveled in malaria-endemic areas	No data	No data	No data	No data	No data	*P. falciparum*	No data	Survived	[14]
			No data	No data	No data	No data	No data	*P. falciparum*	Preemptive therapy	Survived	
United Kingdom	Parenteral transmission within the infectious disease unit (IDU) had stayed on the same ward as a man with *P. falciparum*	No data	41-year-old woman	No data	Fever (40 °C), rigors and intermittent diarrhoea, anemia, thrombocytopenia	No data	Rok/9 days	*P. falciparum* (7.9%)	Intravenous quinine (initial loading dose of 1.2 g, then 600 mg three times a day)		[34]
Germany	Donor-derived infection	Cameroon	Turkey	No data	No data	No data	42 days	*P. vivax*	No data	Survived	[35]
India	Donor-derived infection, recrudescence or bite of mosquitoes	No donor information	11 patients	No data	Regular (*n* = 8) and irregular fevers (*n* = 2), afebrile (*n* = 1); severe hemolysis and acute renal failure (*n* = 1); acute vascular rejection (*n* = 1); thrombocytopenia (*n* = 4)	Peripheral blood smear	20–83 days	*P. falciparum* (*n* = 6), *P malariae* (*n* = 1), *P. vivax* (*n* = 1), mix *P. vivax* + *P. falciparum* (*n* = 3)	Chloroquine + primaquine (*P. vivax*), *P. falciparum* I—chloroquine (*P. falciparum* and *P. malariae*)	Survived (*n* = 10), died (*n* = 1)	[12]
Turkey	Reactivation of the disease (minor possibility) or transmission from that malaria-complicated patient	No donor information	Women with possible chronic rejection 24 months after the operation	No data	Fever	No data	24 months	*P. falciparum*	No data	Survived	
India	Donor-derived infection, recrudescence or bite of mosquitoes	Details were unavailable	Taiwan	No data	No data	No data	22 days	*P. vivax*	No data	Survived	[36]
			Taiwan	No data	No data	No data	29 days	*P. ovale*	No data	Survived	
India	Donor-derived infection	25-year-old Indian man	33-year-old man with chronic renal disease from Turkey	No data	Fever	Peripheral blood smear	30 days	*P. vivax*	Chloroquine	Survived	[37]
France	Donor-derived infection	51-year-old Cameroonian woman	45-year-old man	No data	No data	Serological tests for malaria antibody	30 days (weakly positive for antigen)	No data	No data	Died 45 days after transplantation (cytomegalovirus infection)	[38]
			4-year-old boy	No data	No data	Serological tests for malaria antibody	Negative results 105 days after transplantation	No data	No data	Survived	
Switzerland	Donor-derived infection	27-year-old man from Zaire	28-year-old Swiss man with glomerulonephritis	No data	Fever with irregular peaks	Peripheral blood smear	9 days post-Tx	*P. vivax*	Chloroquine	Survived	[39]
			29-year-old Swiss man with glomerulonephritis	No data	Fever with peaks every 48 h	Peripheral blood smear	16 days post-Tx	*P. vivax*	No data	Survived	
England	Donor-derived infection, recrudescence	His brother, 25 years old and in good health, from Jizan	27-year-old Saudi man	No data	Aching in limbs, chest, occasional rigors, and a daily fever up to 38.5 °C	Blood peripheral smear	39 days post-Tx	*P. falciparum*	Chloroquine	Survived	[40]
United States	Donor-derived infection, recrudescence or transfusion of blood	43-year-old mother from Ghana	27-year-old woman from Ghana had lived in USA	No data	Fever, AKI, aching limbs	Blood smear	35 days post-Tx	*P. falciparum*	Chloroquine	Survived	[41]
United States	Donor-derived infection, recrudescence or transfusion of blood	His brother, 18-year-old from Nigeria	28-year-old man with hypertension and azotemia from Nigeria	No data	Fever (38 °C), chills, and a dry cough, oliguric	Blood smear, indirect immunofluorescent test	33 days/9 days	*P. malariae*—blood smear, *P. falciparum*—immune test (1:64)	Primaquine	Survived	[42]
**Heart**											
Colombia		22-year-old male patient with a gunshot wound to the brain and brain death from Venezuela	A 29-year-old female patient with a history of Hodgkin’s type lymphoma	Cyclosporine, mycophenolate mofetil, and prednisone	Tachycardia, hypotension, anemia, and fever	Peripheral blood smear, PCR	10 days/30 days	*P. vivax*	Artesunate (2.4 mg/kg) for 24 h and chloroquine and primaquine for 14 days	No data	[2]
Colombia		Young man who suffered a severe penetrating traumatic brain injury caused by a gunshot wound, who was otherwise healthy	34-year-old Colombian obese woman with advanced heart failure	No data	Fever, cephalalgia and tonic–clonic seizures with MRI findings compatible with posterior reversible encephalopathy	Thick blood smear	14 days/18 days	*P. vivax*	Daily intravenous artesunate (2.4 mg/kg) was initiated for 3 days with 14 days of maintenance treatment with chloroquine and primaquine	Died	[15]
Spain (heart)	Infected donor	34-year-old male from Mali, who died from head trauma; a peripheral blood smear to exclude malaria—negative	50-year-old Spanish man with idiopathic dilated cardiomyopathy	No data	Fever of 38.5 °C, chills, and hypotension	Peripheral blood smear	15 post HT/17 post HT	*P. falciparum* (parasitemia rate of12%)	Quinine, doxycycline, artesunate	Survived	[17]
Iran	Donor-derived infection	Iran	Iran	No data	No data	No data	10 days	*P. falciparum*	No data	Survived	[43]
Spain	Donor-derived infection	Born in Bolivia, lived in Columbia	No data	No data	No data	No data	No information	*P. vivax*	No data	Survived	[31]
Germany	Donor-derived infection	Cameroon	Germany	No data	No data	No data	No information	*P. vivax*	No data	Survived	[35]
France	Donor-derived infection	51-year-old Cameroonian woman	53-year-old Portuguese woman for left ventricular Insufficiency (Heart)	No data	Fever of 40 °C	Giemsa-stained thin smear	12 days post-admission/18 days post-transplant	*P. falciparum*	Intravenous quinine 20 mg/kg/day	Died of complicated malaria on day 22.	[38]
		Not available/Saudi Arabia	No details available		Second kidney transplantation	Thrombocytopenia, graft failure, acute vascular thrombosis		2% *P. vivax*	Artesunate, followed by primaquine		[11]
France	Donor-derived infection	Man from Togo, often traveled in malaria-endemic areas	46-year-old man for end-stage cardiomyopathy from France	No data	Fever, neurological disorders, acute renal failure with severe acidosis, cytolysis, anemia, thrombocytopenia	No data	No information	*P. falciparum* (30%)	No information	Died-multiple organ failure syndrome (17 days po transplantacji)	[14]
Republic of China	Donor-derived infection	Unknown donors in India	24-year-old male, a native of Taiwan with chronic renal failure due to nephrotic syndrome	No data	Fever	Peripheral blood	18 days	Ring forms, schizonts and trophozoits of *P. vivax*	Chloroquine	Survived	[44]
**Liver**											
Columbia	Donor-derived infection	From Montería (a malaria-endemic area in northern Colombia)	54-year-old man with cirrhosis from Colombia—liver	Prednisolone (20 mg once a day), azathioprine (150 mg once a day), tacrolimus (3 mg twice a day), and aspirin (100 mg once a day)	Malaise, chills, fever (up to 39 °C), dysuria, tenesmus	Thick blood smear	30 days	*P. vivax* (2280 parasites/uL)	Arthemeter/lumefantrine, relapse after 2 months—chloroquine + primaquine	Survived	[6]
	Bite of mosquitoes or transplanted organ	Colombia/Córdoba, Colombia	58-year-old woman from Córdoba	Prednisolone (5 mg once a day), tacrolimus (1 mg twice a day), and mycophenolate mofetil (1000 mg twice a day)	Malaise, fever, chills, vomiting, arthralgia (mainly in the knees), headache, and lumbar pain	Thick blood smear	6 months post-Tx	*P. vivax* (50,640/uL)	Chloroquine (three days) and primaquine (14 days)		
France	Transmission by liver transplantation	51-yearold man from Ghana who had arrived in Spain	51-year-old Spanish man with giant cavernous hemangioma	Tacrolimus, mycophenolate mofetil, and corticosteroids	Fever (40 °C), general malaise, headache and chills, thrombocytopenia	Smear was performed by a Sysmex SP-50™ automated system, using a May-Grünwald Giemsa stain, rapid immunochromatographic assay, and real-time PCR	27 days	*P. malariae*	F piperaquine/dihydroartemisinin 320 mg/40 mg	Survived	[13]
Colombia	22-year-old male patient with a gunshot wound to the brain and brain death from Venezuela	No data	A 50-year-old man	Cyclosporine, azathioprine, and corticosteroids	Severe thrombocytopenia, myalgia, arthralgia, and general discomfort	Peripheral blood smear	20 days/30 days	*P. vivax* (100,808 forms/µL)	Chloroquine 600 mg, at 24 and 48 h chloroquine at a dose of 450 mg; primaquine 15 mg/day for 14 days	Survived	[2]
Italy	Transplant tourism in India, 8 months in India, returned to Sierra Leone for 4 months, then traveled to Italy	Living related donor (Mother, asymptomatic carrier)	2-year-old male with biliary atresia from Sierra Leone	No data	Malaise, fever, anemia	Light microscopy of stained blood films	7th day of hospitalisation	*P. falciparum*, (10%)	Artesunate i.v. 3 mg/kg for 3 days (at 0, 12, 24, 48, 72 h), followed by a course of oral artemether/lumefantrine (20 mg/120 mg at 0 and 8 h, then every 12 h for the other 2 days)	No data	[21]
Colombia	No data	Young man who suffered a severe penetrating traumatic brain injury caused by a gunshot wound, who was otherwise healthy	51-year-old man	No data	No data	Peripheral blood smear	No data	*P. vivax* (10,080 forms/µL)	No data	Survived	[15]
Spain (liver)	Donor-derived infection	Mali	No information, an asymptomatic liver recipient	No data	No data	Peripheral blood smear, Serological Stests for malaria antibody	No information	*P. falciparum*	Quinine sulfate and doxycycline	Survived	[17]
India	India	50-year-old woman with subarachnoid hemorrhage	30-year-old man for cryptogenic cirrhosis	Tacrolimus, mycophenolate mofetil, and corticosteroids	Fever, chills, enlarged spleen, anemia	Peripheral blood film, Immunochromatographic test	44 days	*P. falciparum*, *P. vivax*	Quinine sulfate (10 mg/kg 8 hourly infusion in 5% dextrose for 3 days, then 600 mg orally three times per day for 7 days) and artemether 3.2 mg/kg intramuscularly as loading dose followed by 1.6 mg/kg/day intramuscularly for 5 days	No data	[45]
Singapore	Donor-derived infection, recrudescence, mosquito Bite, blood transfusion	Her nephew from Myanmar	65-year-old woman from Myanmar, resident of Singapore, with Underlying hepatitis C cirrhosis	Basiliximab (on Days 0 and 4), tacrolimus and mycophenolate sodium, with everolimus being added 2 weeks later	Fever	Peripheral blood examination	32 days	*P. vivax* (0.5%)	Mefloquine for 3 days Followed by primaquine for 14 days	Survived	[46]
		Nephew from India	53-year-old diabetic man from Mumbai (India) with alcoholic cirrhosis	Tacrolimus and prednisolone	Pneumonia, prolonged Ventilation, hyperbilirubinemia, fever	Peripheral blood smear	19 days	*P. vivax* (0.5%)	Chloroquine for 3 days, followed By primaquine for 2 weeks	Survived	
Spain	No data	27-yr-old male From Colombia, history of malaria	30-yr-old male	No data	Thick blood smear	Fever with chills and hypotension	21 days	*P. vivax*	Chloroquine (total dose: 1500 mg) and a double dose of primaquine (30 mg/day for 14 days, total dose: 420 mg);	No data	[31]
Columbia	Donor-derived infection, recrudescence	25-year-old Afro-Colombian man from Medellín, Colombia	46-year-old woman from Colombia with sudden liver failure of unknown cause	Azathioprine, methylprednisolone, cyclosporine, and daclizumab	Fever (39 °C), chills, pontine myelinosis, anemia	Peripheral blood smear	22 days post-admission/30 days post-transplant	*P. vivax*	Chloroquine for 3 days (1.5 g, total dose) and primaquine (15 mg/d) for 14 days (210 mg, total dose)	Survived	[47]
Italy (liver)	Donor-derived infection	30-year-old man from Ghana	64-year-old man with acute liver failure of undetermined etiology	No data	Multi-organ failure, fever (40 °C)	Peripheral blood smear	30 days	*P. falciparum* (10%)	Intravenous quinine sulfate (20 mg/kg loading dose, followed by 10 mg/kg 3 times per day), oral doxycycline (100 mg twice per day)	Survived	[32]
France	Donor-derived infection	Man from Togo, often traveled in malaria-endemic areas	54-year-old man with posthepatitis C cirrhosis from France	No data	Thrombopenia, twofold cytolysis, alteration of neurological status	Peripheral thick blood smear	8 days	*P. falciparum* (15%)	Quinine 25 mg/kg/day, vibramycin	Survived	[14]
Germany	Donor-derived infection	Cameroon	Germany	No data	No data	No data	35 days	*P. vivax*	No data	Died	[35]
France	Donor-derived infection	Resident of Ivory Coast	45 from Italy	No data	Fever, neurological disorder		33 days czy 22 days	*P. falciparum*	Quinine	Died	[48]
**Lung**											
France	Donor-derived infection	Lived in Africa for several years; pre-transplant malaria PCR negative	63-year-old woman	No data	Anemia, thrombocytosis, pain in the right hypochondrium	PCR was negative before transplantation	14 days	*P. ovale*	Not reported	Severed	[16]
Spain	Donor-derived infection	Mali	No information, bilateral-lung recipient	No data	Fever	Peripheral blood smear	No information	*P. falciparum*	Quinine sulfate and doxycycline	Survived	[17]
